# Validation of a dermatologic surface area smartphone application: EZBSA

**DOI:** 10.1111/srt.13118

**Published:** 2021-12-19

**Authors:** Conor Vickers, Jalal Maghfour, Indermeet Kohli, Henry W. Lim, Iltefat H. Hamzavi

**Affiliations:** ^1^ Department of Dermatology, Lewis Katz School of Medicine Temple University Philadelphia Philadelphia Pennsylvania USA; ^2^ Department of Dermatology Henry Ford Hospital Detroit Michigan USA

To the Editor:

Precise assessment of the surface area involvement of vitiligo as well as other dermatologic conditions is an important part of clinical research and treatment. Surface area measurements of vitiligo can reveal clinically significant changes that can affect management of patients. Previously investigated reports have demonstrated the validity of computer‐based software programs in the measurement of vitiligo‐affected surface area and studies investigating the variability in measuring the three‐dimensional nature of skin lesions.^1^, ^2^ In this study, we sought to evaluate the accuracy and reliability of a novel technique, using a smartphone application (SA): EZBSA, in measuring body surface area.

**FIGURE 1:**
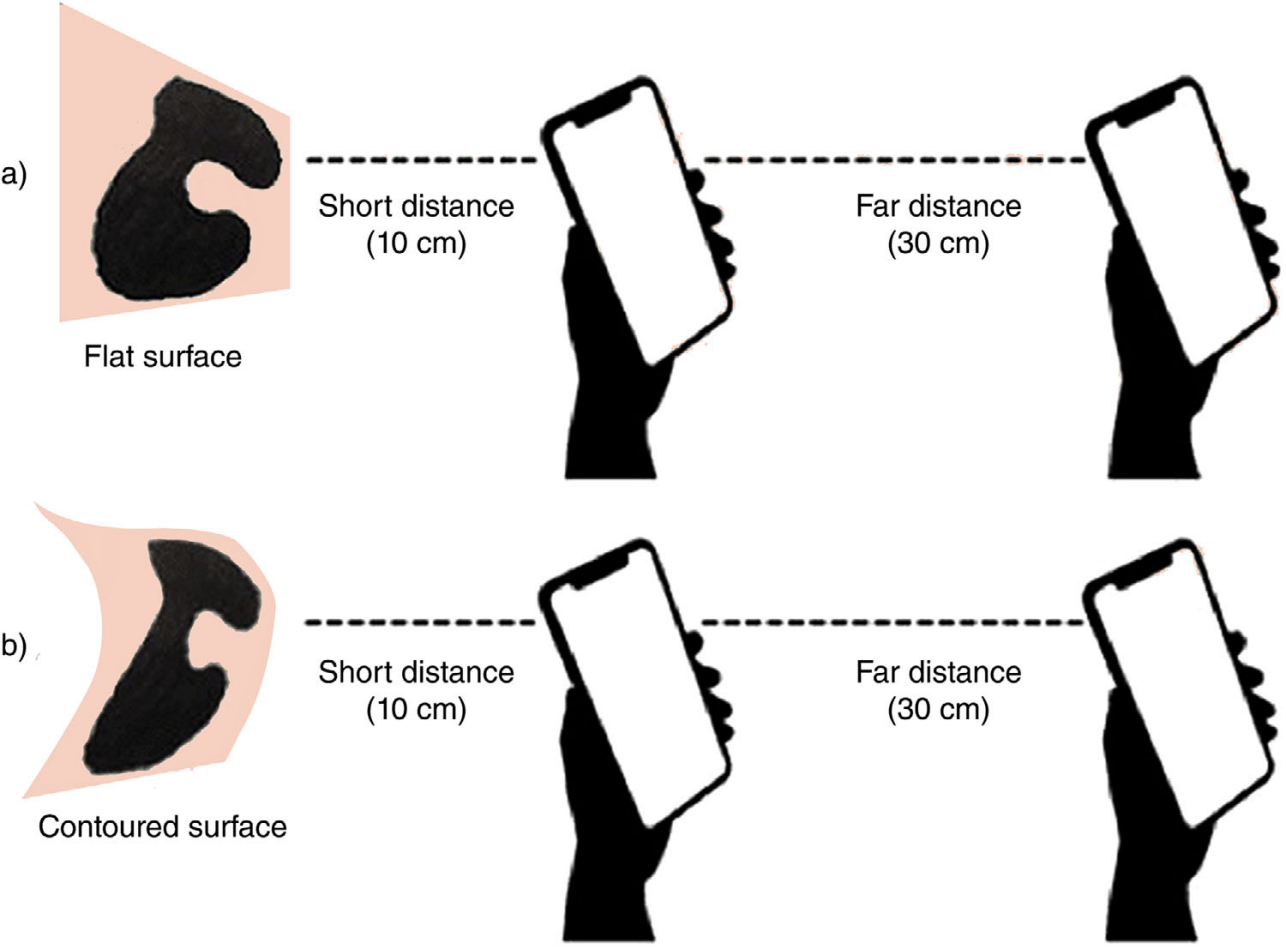
Representation of experimental setup for near and far distances for (A) flat and (B) contoured surfaces using abstract shape 7 as an example

Using EZBSA, images were obtained of 10 different shapes of known dimensions from a short distance (10 cm) and a farther distance (30 cm) by placing the shape on both a flat surface and on the forearm (Figure 1A,B). When placed on the forearm, the shapes were flushed to the natural curvature of the skin (Figure 1B). Three of the 10 shapes were chosen to have well‐defined geometry (square, circle, triangle), and the remaining seven had abstract shapes with width ranging from 2 to 7 cm. The abstract shapes were chosen to simulate skin lesions. Surface area measurements were made with both EZBSA app and ImageJ software (Table 1). Pearson correlation analysis was used to evaluate the agreement between the measurements. In addition, the paired *t*‐test was performed to evaluate an upward or downward shift in the measured areas as detected by the EZBSA app compared to that by imageJ software. A statistically significant (*p* < 0.001) Pearson correlation coefficient was found between the EZBSA app‐ and imageJ‐measured surface areas from the images acquired at 10 cm (0.995) and 30 cm (0.981) away. Paired *t*‐test results indicated no statistically significant differences (*p* > 0.05) between the smartphone app‐ and imageJ‐measured surface areas (Table 2).

**TABLE 1:** Assessment of body surface area using EZBSA^™^ and ImageJ

		EZBSA (cm^2^)		ImageJ (cm^2^)		Geometric calculation
Surface	Shape	Near picture	Far picture	Near picture	Far picture	
Flat surface	Square	40.990	45.765	39.172	43.124	40.322
	Circle	30.998	30.535	31.874	30.513	31.669
	Triangle	21.023	19.096	20.559	21.794	20.161
	Abstract 1	16.305	15.475	15.508	14.663	–
	Abstract 2	3.524	4.280	3.206	2.781	–
	Abstract 3	29.936	30.448	31.279	34.771	–
	Abstract 4	19.878	22.990	20.239	22.836	–
	Abstract 5	15.825	16.174	14.473	14.769	–
	Abstract 6	15.259	16.254	13.486	13.435	–
	Abstract 7	10.414	13.359	9.733	9.908	–
Forearm	Square	39.580	38.162	30.521	40.871	40.322
	Circle	27.275	29.329	26.296	27.698	31.669
	Triangle	17.652	18.705	17.094	19.508	20.161
	Abstract 1	13.577	15.767	12.732	15.785	–
	Abstract 2	2.758	3.388	2.773	3.260	–
	Abstract 3	30.806	30.806	30.488	31.267	–
	Abstract 4	17.295	20.534	18.670	19.024	–
	Abstract 5	15.024	16.034	15.150	15.404	–
	Abstract 6	14.408	13.416	13.848	13.609	–
	Abstract 7	9.940	8.831	8.595	9.786	–

**TABLE 2:** Surface area measurement results for EZBSA^™^ smartphone application and imageJ software, at near (10 cm) and far (30 cm) distances on flat and forearm surfaces

**Correlation**	**Pearson correlation coefficient**	***p*‐Value**
EZBSA app vs. ImageJ: near distance	0.995*	<0.001
EZBSA app vs. ImageJ: far distance	0.981*	<0.001
EZBSA app near image vs. far distance	0.985*	<0.001
ImageJ near image vs. far distance	0.993*	<0.001
Paired *t*‐test	Mean ± SEM	*p*‐Value
EZBSA app vs. ImageJ: near distance	App: 20.42 ± 3.46 ImageJ: 19.95 ± 3.52	>0.05
EZBSA app vs. ImageJ: far distance	App: 21.44 ± 3.67 ImageJ: 20.86 ± 3.89	>0.05
EZBSA app near image vs. far distance	Near: 20.42 ± 3.46 Far: 21.44 ± 3.67	>0.05
ImageJ near image vs. far image	Near: 19.95 ± 3.52 Far: 20.86 ± 3.89	>0.05
EZBSA app near flat vs. contoured	Flat/2D: 20.42 ± 3.46 Contoured: 18.83 ± 3.41	<0.05
EZBSA app far flat vs. contoured	Flat/2D: 21.44 ± 3.67 Contoured: 19.49 ± 3.35	<0.05

The asterisk indicates statistical significance.

When comparing the measurements from the smartphone app for the shapes on flat surfaces and contoured surfaces, a statistically significant (*p* < 0.001) Pearson correlation coefficient was found for both near (0.991) and far (0.978) distances. A paired *t*‐test between the measurements from the smartphone app, on flat and contoured surfaces, indicated statistically significant differences (*p* < 0.05) for both near and far distances, with area measurements from pictures of the shapes acquired at contoured surface being approximately 10% lower than the corresponding measurements from flat surface (Table 2).

A high correlation coefficient between the smartphone app measurements and ImageJ measurements imply excellent agreement between both techniques. Of note, there was less than 1% error when comparing app measurements of two‐dimensional flat images of known geometries to the corresponding known/calculated areas (Table 1).

Despite a high correlation, the results of paired *t*‐tests indicated that area measurements from contoured surfaces were approximately 10% lower compared to corresponding flat surface measurements. This is expected as pictures from contoured surface are two‐dimensional projection of a three‐dimensional surface. These findings are consistent with previously reported discrepancies between area measurements from two‐ and three‐dimensional photos.1^,^2 Limitations of this study include the use of simulated lesions with a limited range of sizes and a single unblinded app user. In addition, this SA was not used in a clinical setting, and thus future studies are needed to assess actual lesions in clinical studies and evaluate corresponding reliability and reproducibility.

In summary, the results show that EZBSA is a simple, reliable, accurate, and valid alternative to perform surface area measurements. For accuracy, pictures are recommended to be taken in a well‐lit room with the smartphone aligned parallel to the lesion, and care should be taken to accurately trace lesion border on the acquired images.
